# Typhoid Fever

**Published:** 1856-03

**Authors:** 


*Typhoid Fever.*—Dr. Shute, of the Torbay Infirmary, treated during the year 1854, forty-eight cases of typhoid fever in that institution, losing but one patient. His treatment as recorded in the Medical Times and Gazette, was to use brandy and quinine freely, and sustain and nourish the patient. The doctrine of feeding fevers is becoming generally approved of, and the success in the results of the treatment are encouraging.

